# Fundamental and practical aspects of machine learning for the peak picking of biomolecular NMR spectra

**DOI:** 10.1007/s10858-022-00393-1

**Published:** 2022-04-07

**Authors:** Da-Wei Li, Alexandar L. Hansen, Lei Bruschweiler-Li, Chunhua Yuan, Rafael Brüschweiler

**Affiliations:** 1 Campus Chemical Instrument Center, The Ohio State University, Columbus, OH 43210 USA; 2 Department of Chemistry and Biochemistry, The Ohio State University, Columbus, OH 43210 USA; 3 Department of Biological Chemistry and Pharmacology, The Ohio State University, Columbus, OH 43210 USA

**Keywords:** NMR spectroscopy, Machine learning, Deep learning, Deep neural network, Peak picking, Spectral analysis

## Abstract

Rapid progress in machine learning offers new opportunities for the automated analysis of multidimensional NMR spectra ranging from protein NMR to metabolomics applications. Most recently, it has been demonstrated how deep neural networks (DNN) designed for spectral peak picking are capable of deconvoluting highly crowded NMR spectra rivaling the facilities of human experts. Superior DNN-based peak picking is one of a series of critical steps during NMR spectral processing, analysis, and interpretation where machine learning is expected to have a major impact. In this perspective, we lay out some of the unique strengths as well as challenges of machine learning approaches in this new era of automated NMR spectral analysis. Such a discussion seems timely and should help define common goals for the NMR community, the sharing of software tools, standardization of protocols, and calibrate expectations. It will also help prepare for an NMR future where machine learning and artificial intelligence tools will be common place.

## An old and hard problem: computer-assisted NMR peak picking

A critical step in the analysis of complex NMR spectra of proteins, RNA, DNA and molecular mixtures is the identification of individual peaks in 1D and cross-peaks in 2D and higher dimensional NMR spectra. Peak identification, often referred to as peak-picking, is a prerequisite for all subsequent steps in spectral analysis and interpretation, including resonance assignment and peak quantitation for biomolecular interaction and dynamics studies or the unambiguous elucidation of the composition of complex mixtures. Computer-assisted and automated 2D NMR peak picking has a long history starting in the 1980s shortly after the introduction of 2D NMR (Neidig et al. 1984). However, the development of peak picking algorithms that can deal with strong spectral overlap and the presence of spectral artifacts has proven challenging to this day. Traditional peak pickers examine spectra in terms of geometric properties, (Pfandler et al. 1985; Meier et al. 1987; Bartels et al. 1995; Koradi et al. 1998; Johnson 2004; Garrett et al. 1991; Liu et al. 2012; Skinner et al. 2016; Wurz and Güntert 2017) such as local maxima, local symmetry, contour line features, curvature and related features, or utilize matrix factorization, (Korzhneva et al. 2001; Orekhov et al. 2001; Tikole et al. 2014) singular value decomposition Alipanahi et al. (2009) and multivariate Gaussian densities, (Antz et al. 1995; Rouh et al. 1994; Cheng et al. 2014) which allows the reliable identification of individual peaks, but only if they are sufficiently separated from each other. Other methods directly combine time-domain fitting and peak picking (Krishnamurthy 2013; Krishnamurthy et al. 2017; Hansen and Brüschweiler 2016; Li et al. 2018; Hansen et al. 2017). On the other hand, these peak pickers are challenged when presented with spectral regions containing peak pairs or entire peak clusters that show high levels of crowding, a high dynamic range, or shoulder peaks that are overshadowed by strong neighboring peaks. Spectra with such features commonly occur for complex molecular systems, such as large proteins, protein–protein and protein-DNA/RNA complexes, intrinsically disordered proteins, or complex metabolomics mixtures.

Over the past few years, dramatic progress in machine learning (ML), in particular the advent of deep neural networks (DNN), has opened new opportunities and led to powerful new tools to tackle this long-standing challenge. This technology has been made broadly accessible by means of DNN frameworks, such as TensorFlow (Abadi et al. 2016) and PyTorch (Paszke et al. 2017). Most recently, we trained a deep neural network, called DEEP Picker, for the accurate analysis of 2D NMR spectra of challenging protein and metabolomics samples (Li et al. 2021). During this project, a series of fundamental questions came into focus, such as what constitutes reasonable expectations for a peak picker, what role machine learning can play, how peak-pickers should be trained, how their performance should be assessed, and whether there are limits for the automation of peak picking. The purpose of this perspective is to address these and other questions along with illustrations and suggestions with the hope to stimulate fruitful discussions about these issues also within the wider NMR community.

As for other areas of the natural sciences, it is expected that ML and related developments in artificial intelligence (AI) will play an increasingly important, if not dominant, role for the future of automated data analysis and interpretation. Therefore, this perspective may be of interest for developers and end users of such software alike. Each of the following sections addresses a particular objective or challenge.

## A new era for NMR spectral analysis by machine learning

**Fig. 1:**
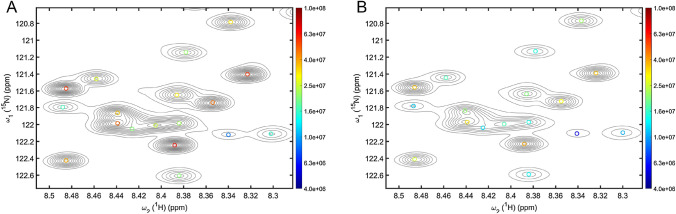
Crowded region of 2D ^15^N–^1^H HSQC spectrum of α-synuclein using **A** uniformly sampled time-domain data and **B** non-uniformly sampled (25%) time-domain data with spectral reconstruction using SMILE. Contour lines are plotted using a linear scale and the cross-peaks picked by the DNN (DEEP Picker) are indicated by open circles that are color-coded according to the cross-peak amplitudes (logarithmic scale, see color sidebar). The uniformly sampled spectrum was collected with 256 × 1024 complex points and zero-filled to 2 K (N_1_) × 8 K (N_2_) points. The results returned by the DNN for the uniformly sampled and NUS spectra are interchangeable (see also main text)

The basic premise of a machine-learning based NMR peak picker is that it is possible to train an algorithm with a database of NMR spectra of known NMR peak composition so that it then can be applied for the accurate and comprehensive identification of NMR signals that were not used for training. This idea is not new. In fact, early attempts using artificial neural networks for pattern recognition in 1D and 2D NMR spectra date back to the late 1980s and early 1990s (Thomsen and Meyer 1989; Carrara et al. 1993). However, over the ensuing decades, ANNs could not establish themselves as standard tools against more traditional spectral analysis methods. The task of peak recognition in NMR spectra loosely resembles the object recognition problem in photographs and images, a burgeoning field enabled by gigantic image databases generated in part through social media. Much of the recent progress in image recognition stems from major developments in the theory and practical implementation of DNNs, especially for the subclass of convolutional neural networks, which greatly benefit from vast improvements in computer power available today. Therefore, we will focus here on machine-learning approaches based on DNNs although many of the considerations apply also to other machine-learning methods [for a general review, see Alpaydin 2020]. It should be noted that the DNN returns peak positions with peak shapes and amplitudes whose accuracy depends, among other factors, on the degree of overlap. Figure 1A illustrates the performance of a DNN when applied to a crowded region of a ^15^N–^1^H HSQC spectrum of α-synuclein. The results returned by the DNN correctly recover the true number of peaks with their true positions that were independently determined from a standard suite of 3D NMR experiments of a uniformly ^13^C,^15^N-labeled sample.

## General considerations for experimental vs. synthetic spectral training databases

In any machine-learning project, the importance of the quality and size of the database used cannot be understated. The ideal database should cover a sufficiently large number of spectra that contain NMR peaks of different amplitudes and shapes resembling those expected in target applications. Moreover, the database spectra should encompass the fullest possible range of peak configuration scenarios ranging from peaks that are well isolated to strongly overlapping peak clusters along with a large dynamic range of peak amplitudes and peak widths. Such a database will then allow the training of a DNN for the picking of individual peaks of both well-resolved and crowded spectral regions. The underlying assumption is that a more comprehensive representation of peak properties and configurations in the training database will improve the performance of the DNN peak picker when applied to spectra in real-world applications.

For the development of a machine-learning based peak picker for 2D or higher dimensional NMR spectra, the training set ideally consists of a vast amount of experimental 2D NMR spectra, e.g. ^15^ N–^1^H HSQC, NOESY, and TOCSY spectra, of many different protein systems that were recorded with similar acquisition parameters and processed in the same way. These data sets will then allow the training for the identification of isolated peaks and peaks that show a variable degree of overlap using assignments made by human experts. Such a strategy was chosen in the original application of neural networks to peak picking (Carrara et al. 1993) and also in the recent NMRNet approach (Klukowski et al. 2018) for the automated analysis of NOESY-type spectra. A related approach has been proposed to filter out noise peaks to facilitate the automated analysis of multidimensional datasets (Kobayashi et al. 2018). The challenges are that such a large collection of experimental spectra needs to be carefully curated, including uniform experimental parameters and processing, and that even a large number of experimental spectra used in this way will only sample a subset of possible peak overlap scenarios, especially for peak clusters that involve three or more cross-peaks. This carries the risk that the neural network may be inadequate for the deconvolution of crowded regions consisting of sets of peaks that mutually overlap or overlapping peaks with large amplitude or linewidth differences, since they may be too different from the scenarios covered by the training database. Such deficiencies and gaps in experimental training sets are difficult to spot and may only be revealed during the application phase of a DNN. Another potential complication with an experimental training database is that the correct decomposition of a spectral region into the sum of true individual resonances may be unknown or only partially known. It should also be kept in mind that a feature annotated as a “true peak” by a human expert based on visual inspection, does not always guarantee that it is in fact real.

As elsewhere in machine learning, the *ground truth* describes the reality one wants the model to accurately predict. Part of the ground truth information about experimental NMR peaks, such as peak positions of 2D ^15^ N–^1^H or ^13^C–^1^H HSQC peaks, can often be obtained from higher dimensional NMR spectra of isotopically labeled proteins that exhibit minimal peak overlap, whereas the true width and volume of each individual peak may not be easily obtainable by experiments for strongly overlapped peaks. By contrast, in the case of NOESY-type experiments the reality of a peak, especially when it is weak, is often hard to prove as it would require the highly accurate knowledge of a biomolecule’s conformational ensemble in solution and its dynamics time scales, which are only rarely available.

Many of these issues can be avoided by choosing an entirely synthetic training database. In this case, the ground truth of each database entry is the set of all individually simulated peaks, prior to their summation, for which all peak parameters (position, width, volume) are precisely known. The advantage of synthetic databases is that the ground truth is always known without ambiguities. Synthetic databases also allow the generation of densely sampled spectral sets that adequately represent the many possible complex peak overlap situations, including ones that may be missed in experimental databases.

**Fig. 2:**
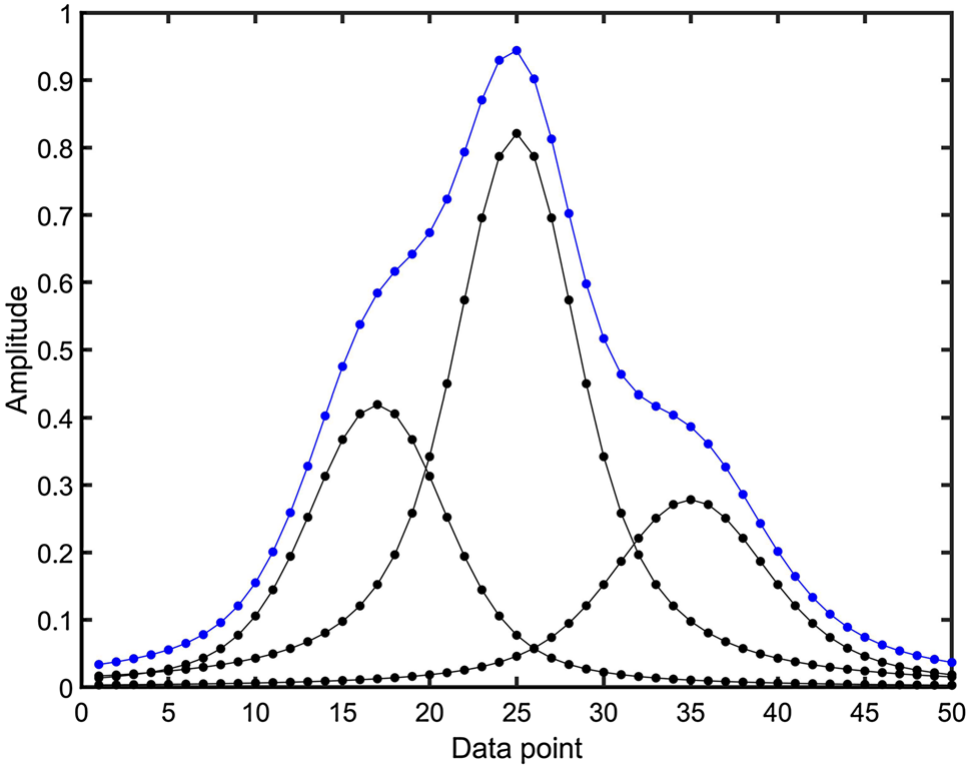
Example of a synthetic spectrum used for training of DNN peak picker. The blue spectrum represents the superposition of 3 individual overlapping Voigt peaks (black peaks). The blue sum spectrum together with the 3 individual Voigt peaks (ground truth) serve as input for the training of the DNN peak picker

Figure 2 shows an example of a synthetic 1D spectrum consisting of three individual overlapping peaks, which represent the ground truth and are suitable to be part of the training of the DNN peak picker. Before its use for training, however, it is important to curate a synthetic spectral database by removing entries whose peak overlaps are too strong and that are virtually impossible to unambiguously deconvolute in the presence of noise. Removal of such entries prevents feeding the neural network with ambiguous training data resulting in poor performance. As a third option, one could use a hybrid spectral database for DNN training that contains both automatically annotated synthetic spectra and manually annotated experimental spectra whereby the relative weights of the two types of databases can be optimized during training.

A synthetic database for training and validation can be directly computed in the frequency domain by the co-addition of generated peaks with randomly chosen positions, lineshapes, and volumes as done for DEEP Picker. Conversely, peaks can be simulated in the time-domain, followed by zero-filling, apodization, and Fourier transformation. The latter method allows the simulation of peak shapes that closely resemble those found in actual NMR experiments. In addition, Gaussian random noise can be added to the time domain data with a standard deviation, e.g., as a constant fraction of the volume of the largest peak. Furthermore, it is possible to introduce systematic artifacts similar to those encountered in practice, such as small phase errors and baseline offsets. However, with the inclusion of more “degrees of freedom”, the database will need to become larger to adequately represent the combined presence of these effects in the database spectra. In the case of DEEP Picker, it turned out that the use of training spectra directly simulated in the frequency domain without the explicit inclusion of the above-mentioned artifacts (noise, phase, baseline) was sufficient.

When generating a synthetic spectral database, information about peak shapes to be expected in subsequent applications is important allowing a more targeted training of the DNN to improve peak-picking accuracy. In solution NMR spectra, peak shapes are in good approximation a convolution of Lorentzian and Gaussian lineshapes, which is a consequence of the exponentially decaying free induction decay (FID) subject to apodization with commonly used window functions, such as a shifted sine-bell, cosine square or 2π-Kaiser window, prior to zero-filling and Fourier transformation. The convolution of Lorentzian and Gaussian lineshapes is known as Voigt profile *V*(ω), which can be expressed as the real part of the complex Fourier transformation of functions of the type exp(iω_0_*t*-*R*_2_*t*-*bt*^2^):

1 $$V(\omega ) = Re\int\limits_{0}^{\infty } {Ae^{{i\omega_{0} t - R_{2} t - bt^{2} }} } e^{ - i\omega t} dt$$

where *A* is the peak volume, ω_0_ is the resonance frequency defining the peak position, *R*_2_ = 1/*T*_2_ is the transverse relaxation rate, and *b* defines the Gaussian contribution to the lineshape (the lineshape is Lorentzian in the limit of *b* = 0 and Gaussian in the limit of *R*_2_ = 0). In practice, it can be advantageous to use pseudo-Voigt functions (Zaghloul and Ali 2011) for the efficient generation of synthetic spectra. The Voigt lineshape was used for the training of DEEP Picker. Experimental spectra with peak shapes that significantly and systematically deviate from Voigt profiles may be picked less accurately and, hence, they will require training of a peak picker using a different training database with spectra that accurately represent the target peak shapes.

One of the training parameters is the digital resolution of the spectrum compared to the linewidth of a typical resonance. It can be expressed as the number of spectral data *p*oints *p*er *p*eak (PPP) and which is the typical number of spectral datapoints across a peak’s full width at half height (FWHH). In the case of DEEP Picker, peaks should be represented at fairly high density, i.e. PPP should fall into the range from 6 to 20 points or from 4 to 12 points, whereby the former is typical for proteins and the latter for metabolomics spectra. By guaranteeing that an input spectrum has a PPP in the above range, the performance accuracy of the DNN is improved while keeping the size of the spectral training set reasonably small. For real-world applications, the input spectra should have a PPP in the same range as the training datasets. If needed, PPP can be adjusted for a spectrum even after it has been recorded simply by the application of the appropriate amount of zero-filling. It is therefore not necessary to increase the actual spectral resolution via the collection of additional data points at longer t_1_-evolution or t_2_-acquisition times.

## DNN peak picker: training, testing, validation

Peak picking can be trained for 1D, 2D, or higher dimensional spectra. Because in 1D the number of relative peak positions is confined along a single axis, there are fewer possibilities of relative peak arrangements compared to 2D. This allows one to work with a relatively small 1D spectral database to represent relevant configurations defined by the number of peaks, relative peak amplitudes, peak widths, and degree of overlap. Since 2D NMR spectra are defined on a 2D Cartesian frequency grid, there is no rotational symmetry that could be utilized to reduce the database size, although mirror images about the ω_1_ and ω_2_ axes can be exploited. Hence, for a 2D spectral database, many more possible relative orientations of two or more peaks together with their linewidths and peak shapes along the 2nd dimension need to be explicitly represented, which requires a substantially larger database. It is therefore attractive to train a 1D peak picker and subsequently adapt it for peak picking of 2D (or higher dimensional) spectra. The latter can be achieved by separately applying the peak picker to each row and each column and then reconcile the results to obtain the peak positions in 2D or higher dimensions. This strategy can be further refined to avoid false positives for the accurate identification of shoulder peaks as implemented in DEEP Picker (Li et al. 2021). The performance of DEEP Picker is exemplified in Fig. 1 for a ^15^N–^1^H HSQC spectrum obtained both by standard Fourier transformation and non-uniform sampling (NUS) reconstruction using SMILE (Ying et al. 2017). The NUS dataset used 25% of the data points of the conventional dataset with the same t_1,max_. Therefore, the NUS spectrum has around two times lower sensitivity than the 2D Fourier transform (FT) spectrum. Comparing the cross-peak positions (excluding the side-chain NH_2_ groups) of the NUS spectrum by DEEP Picker before and after Voigt fitting with the peak positions of the conventional spectrum (after DEEP Picker and Voigt fitting) yields chemical shift RMSDs of (^1^H:1.2 ppb, ^15^N:17 ppb) and (^1^H:0.84 ppb, ^15^N:15 ppb), respectively. For comparison, the chemical shift RMSD of the conventional spectrum before and after Voigt fitting is (^1^H:0.94 ppb, ^15^N:4.7 ppb). Therefore, the cross-peak accuracy and precision of the NUS and the conventional spectrum achieved by DEEP Picker with or without Voigt Fitter is identical for all practical purposes. Extension of the DNN and Voigt Fitter to 3D and even higher dimensional NMR data is possible and currently under development.

It is quite possible that other strategies produce similar peak-picking performance provided that the training sets and network architectures are suitably chosen for each strategy. For example, models with more degrees of freedom, such as 2D vs. 1D, require significantly larger training databases to prevent overfitting during training. Moreover, the network architecture, including the number of network layers, may need to be adjusted accordingly. Following training, careful *validation*, i.e. the application of the DNN to datasets that are of the same nature as the training datasets but that were not used for training and whose ground truth is known, is critical to assess the performance of any DNN and identify possible overfitting. After successful validation, the DNN undergoes *testing* on a test dataset that is completely independent of both the training and validation datasets to obtain benchmarks of performance.

Once the testing is found satisfactory, the DNN performance is ready to be *assessed* using real-world experimental data. Such an assessment is important to gain confidence in the capability of the DNN in practice. In the case of proteins, suitable test data sets are 2D ^15^N–^1^H HSQC spectra that have been assigned with the help of a standard suite of higher dimensional spectra (such as 3D HNCA, 3D HNCO, etc.). For 2D ^15^N–^1^H HSQC and similar types of spectra a quantitative performance score can be obtained by counting the number of peaks that were correctly identified (true positives), those that were missed by the peak picker (false negatives) and peaks that were identified but may be artifacts (false positives). These numbers can then be converted into normalized standard statistical quantities, such as *precision* = true positives/(true positives + false positives) and *recall* = true positives/(true positives + false negatives) (Ting 2010) to facilitate the comparison of performance of different peak picker software and the monitoring of progress during their further development.

## What can one expect from a neural-network based peak picker?

Although the precision, recall and related measures are conceptually straightforward, their objective evaluation in practice can be tricky. For the determination of true positives, it is possible that two cross-peaks perfectly overlap and the DNN returns only a single peak and, hence, it is counted as one true positive and one false negative. If hydrogen exchange and chemical exchange are not too severe for both underlying cross-peaks, the peak amplitude of this “double peak” is about twice the average peak amplitude, which an experienced NMR spectroscopist will correctly interpret as a “double peak”. By contrast, only if the DNN is instructed to interpret peak amplitude information in such a way, the false negative count can be avoided. Hence, even “flawless” spectral analysis of such a peak by the DNN can lead to an imperfect score.

The situation with false positives is even more complicated as there are several possible reasons why the DNN might identify extra peaks. First, even after purification, NMR samples of proteins are never perfectly homogeneous as there can be residual low-concentration impurities in the sample that give rise to additional cross-peaks. Moreover, protein samples can undergo partial aggregation or degradation that will also cause the presence of low-amplitude cross-peaks. If these additional peaks have amplitudes that exceed the noise floor, there is a good chance that the DNN will pick them leading to a potentially large number of false positives. It should be noted that this is not the fault of the DNN as it is precisely doing what it has been trained for. Because these extra peaks are often relatively weak, it is useful to define a “low peak amplitude cutoff” (LPAC), which is an amplitude threshold below which a peak returned by DNN is discarded. In fact, some kind of cutoff is also used by traditional peak pickers. The LPAC can be defined as a fraction of the amplitude of average true positive peaks or as a multiple of the mean noise amplitude σ_noise_ of the noise floor in a peak-free region of the spectrum, which can be automatically obtained by a robust global noise estimator (Li et al. 2021). The optimal LPAC may vary from protein to protein or even from sample to sample of the same protein as the amounts of impurities and degraded protein may vary and depend on the sample condition, including age as well as storage conditions and measurement temperature. Automatic setting of the LPAC is in principle possible, e.g. by determining the mean amplitude A_mean_ of the *M* largest peaks in a ^15^ N–^1^H HSQC spectrum, where *M* is the number of non-proline residues of the protein sequence, so that

2 $${\text{LPAC = max(A}}_{{{\text{mean}}}} /{\text{X, Y}}\sigma_{{{\text{noise}}}} )$$

where X and Y are scaling factors with a typical range between 5 and 50 depending on the application and the user’s preference. On the other hand, Yσ_noise_ as a lower limit for LPAC, which ensures that the DNN does not pick peaks that are in fact noise features belonging to the noise floor. This definition of LPAC can be directly applied to proteins with different sample concentrations where at high sample concentration LPAC = A_mean_/X and at low concentration LPAC = Yσ_noise_. For the latter case, it is naturally possible that some true positive peaks disappear in the noise floor leading to false negative counts. In the event of substantial line broadening due to rapid H^N^-hydrogen exchange with the water solvent or conformational exchange, certain peaks can be considerably weakened falling below A_mean_/X, thereby also leading to an uptick of the false negative count. Any a priori information available for a given sample and the type of experiment will help one to properly set the LPAC. Fortunately, for resonance assignment experiments of many proteins the performance score of the DNN is not particularly sensitive to the exact LPAC value.

**Fig. 3:**
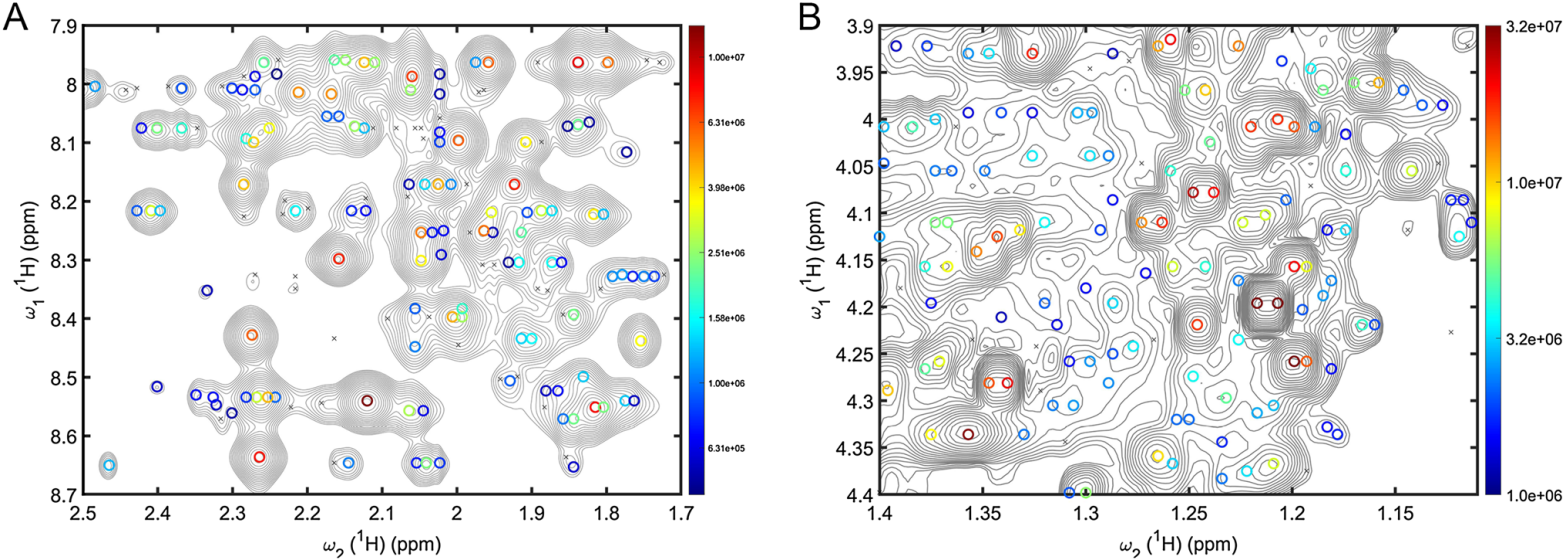
Illustration of the performance of a DNN peak picker for a crowded region of **A** a 2D NOESY spectrum of Im7 protein and **B** a 2D TOCSY spectrum of mouse urine metabolomics sample (contours are plotted on logarithmic scale) together with cross-peak positions returned by DEEP Picker as colored open circles where the colors reflect the peak amplitudes (see color sidebar). Peaks with amplitudes below the low limit of the color sidebar are depicted as gray crosses

Application of DNN peak picking to other types of NMR spectra or samples is of course feasible, such as 2D NOESY, 2D TOCSY, or 2D ^13^C–^1^H HSQC spectra of metabolomics samples (serum, urine, etc.). The quantitative evaluation of peak-picking performance is, however, challenging for such spectra since the ground truth is generally unknown or incomplete. This is due to unassigned protein resonances in NOESY and TOCSY or the presence of unknown metabolites in metabolomics samples. Therefore, peak-picker performance may be best evaluated by human experts visually assessing whether spectral features, including overlaps, are properly deconvoluted by the DNN. Examples of DEEP Picker performance are given for NOESY and TOCSY spectra of a protein and a mouse urine sample in Fig. 3 demonstrating how a DNN is able to handle even highly congested spectral regions. Both NOESY spectra of proteins and metabolomics spectra are well-known for their large dynamic range of cross-peak amplitudes. In the case of a 2D NOESY, highly informative long-range distance cross-peaks have the lowest amplitude due to their r^−6^-internuclear distance dependence, and in metabolomics samples different metabolites can have vastly different concentrations. When setting X in Eq. (2) to a large value, LPAC is solely determined by the noise floor, i.e. LPAC = σ_noise_ where Y can typically take a value in the range of 5–20. This ensures that as many low-amplitude peaks as possible are picked with some carrying important information while others may be discarded during downstream analysis. The lack of ground truth makes such peaks unsuitable for analysis performance scoring. Nonetheless, in certain situations it could be useful to report the number of such picked peaks even if they fall below the LPAC and thus are not considered for downstream analysis. This does not affect the practical utility of the DNN, but it will help to improve our understanding of the inner workings of the DNN and its limitations.

For a given LPAC value, most peak pickers (including DEEP Picker) work in a fully automated mode. Depending on the outcome of the results of downstream spectral analysis, such as resonance assignment or the number of unique metabolites identified, the LPAC value can in principle be iteratively adjusted until the highest level of consistency has been achieved.

## Peak picking in the presence of spectral artifacts

Relevant regions of 2D ^15^ N–^1^H HSQC spectra of proteins are generally “clean” with minimal artifacts, such as t_1_-noise. Also, the residual water signal is up-field shifted with respect to all amide proton frequencies and can therefore be easily excluded before or after analysis by the DNN. By contrast, in 2D TOCSY and NOESY-type spectra, for some resonances the appearance of t_1_-noise is manifested as narrow streaks along their indirect dimension, which may be interpreted by the DNN as a large number of peaks in case that the LPAC is defined by the global noise level. Such false peaks can be filtered out by replacing the global LPAC by one that is increased for regions with pronounced t_1_-noise, although this carries the risk that true but relatively weak cross-peaks in these regions will be missed too.

## Measure of confidence of picked peaks

A quantitative measure of confidence in the output of the DNN for each picked peak is also useful as it can direct users to individual peaks or entire peak regions whose analysis by DNN is potentially ambiguous and challenging. In the case of DEEP Picker, it returns for each spectral point a score for being a major peak, an overlapping minor peak, or not a peak. Peaks that have a low score for being not a peak are assigned a high confidence level, whereas peaks that have a high score for being not a peak are assigned a low confidence level. The latter are also the peaks that are typically hard to deconvolute due to significant overlap or low signal-to-noise (< 10 × σ_noise_). Confidence level information also helps users visually inspect the results by focusing on potentially ambiguous peaks that were earmarked by the software. Traditional peak pickers generally do not return a confidence level for picked peaks. Additional reliability criteria can be included, such as those implemented in iPick, (Rahimi et al. 2021) assessing the volume, linewidth, and signal-to-noise of each peak.

## Application to non-uniformly sampled NMR spectra

For samples and experiments for which spectral resolution is the limiting factor rather than sensitivity, non-uniform sampling (NUS) has become a popular alternative to multidimensional FT NMR (Kazimierczuk and Orekhov 2015). Software packages are available for the spectral reconstruction of NUS data that use different reconstruction algorithms. Because NUS spectra can suffer from artifacts including some that are different from those encountered in Fourier transform NMR (Zambrello et al. 2017), it can potentially cause distortions in spectral lineshapes and the appearance of extra peaks. Figure 1B shows the same ^15^N–^1^H HSQC spectral region used for the demonstration of DNN peak picking, but processed by NUS with a 25% Poisson gap sampling rate using the SMILE software (Ying et al. 2017). The performance of DEEP Picker for this NUS spectrum closely matches that of the fully sampled 2D FT spectrum: when LPAC/σ_noise_ is set to 30 for both the NUS and uniformly sampled spectrum, only three peaks are missing from the NUS reconstruction compared to the fully sampled spectrum due to the inherently lower sensitivity of the NUS spectrum using only a fourth of the time-domain data of the reference spectrum of Fig. 1A. For LPAC/σ_noise_ set to 20 this number is reduced to only one missing peak. These results suggest that DEEP Picker can be readily deployed also to NUS-SMILE spectra without requiring new DNN training. Since different NUS reconstruction software return different results, a systematic analysis for different types of spectra, NUS schedules, and reconstruction algorithms is needed to better understand the potential limitations of a DNN peak picker applied to NUS spectra.

## Short checklist before using DNN-based spectral analysis

The overall workflow of peak picking by a DNN is generally very similar to that of a traditional peak picker. However, a few points need to be considered as summarized here for the case of DEEP Picker in the form of a checklist:

1. The input spectrum should be apodized using a 2π-Kaiser or cosine-square window function along each dimension *without* resolution enhancement. The appearance of Sinc-wiggles should be avoided.
2. The PPP should fall in the range of 4–12 for metabolomics spectra and 6–20 for protein spectra along each spectral dimension. If the PPP of a given spectrum is too low, it can be readily increased by reprocessing the spectrum with adequate zero-filling.
3. Proper phase correction should be applied along all dimensions so that the maximal phase error does not exceed 3°. This is usually easily achievable for spectra acquired on modern NMR spectrometers.
4. Standard baseline correction along all dimensions is advised as implemented in common NMR spectral processing software.

Once being aware of the points listed above, they usually can be fulfilled with little effort for most types of spectra, whereas violation may cause a substandard peak-picking performance that may have an adverse impact on the downstream components of the project.

## Final remarks

With rapid progress in DNNs and other machine learning methods in many areas of science, automated NMR spectral analysis can benefit from these developments when carefully taking into consideration the unique features of NMR spectra. As with traditional peak pickers, the more prior information is available about the NMR sample, be it a biomacromolecular sample or complex mixture, the more accurate and useful will be the output of the DNN peak picker. This includes information about potential artifacts, such as phasing errors, t_1_-noise, baseline distortions or the presence of intrinsically weak cross-peaks, such as Asn and Gln ^15^NH_2_ side-chain cross-peaks in ^15^N–^1^H HSQC spectra. However, even with minimal knowledge about a system for which the NMR spectrum was collected, such as the number of residues of a protein or whether the spectrum stems from a metabolomics sample, DNNs can do a remarkable job in identifying NMR peaks even for spectral regions characterized by extreme crowding and a large dynamic range as was found for DEEP Picker. When paired with automated peak fitting specifically adapted to the DNN output, machine-learning based analysis of NMR spectra offers an advanced degree of automation that helps speed up analysis at improved accuracy. It will make complex spectra amenable to comprehensive analysis, including regions that were previously inaccessible by traditional methods. Despite their unique potential for full automation, for the time being it is still advisable to do quality control by visual inspection at least for part of the spectrum, especially of regions that were earmarked as challenging or ambiguous. Fortunately, DNNs can autonomously identify such “regions of interest” so that human inspection can focus on a relatively small subset of critical cases, thereby minimizing the effort required for obtaining high-quality results in a dependable and reproducible manner. The strategies described here work well for 1D and 2D NMR datasets and we expect that they can be generalized to higher dimensional spectra collected both with uniform and non-uniform sampling schedules. Although we focused here on solution NMR spectra, similar results should be obtainable also for solid-state NMR spectra.

Continuous rapid progress in machine-learning and other AI methodology is likely to soon enable largely automated workflows for NMR data analysis starting from shimming and NMR pulse calibration all the way to the extraction of high resolution, site-specific structural and dynamic data. As magnetic resonance will soon approach its centennial anniversary, this is expected to be yet another milestone in its ever-continuing evolution. It will not only improve both the quality of the output and overall throughput, but also help further broaden the appeal of NMR as one of the most versatile analytical and biophysical techniques making it better accessible to novices and non-experts. Of course, the proper interpretation of all these data in terms of the underlying chemistry, biophysics, biology, and medicine will remain vitally important and eventually determine the impact of these lines of research on their corresponding subfields and beyond. How much machine learning and, by extension, AI at large can assist in this process is currently still open. If the most recent past is a guide, however, it will be a rich and fascinating field to explore with consequential implications for almost every one of NMR’s many facets.
